# Features of Neutrophils From Atopic and Non-Atopic Elite Endurance Runners

**DOI:** 10.3389/fimmu.2021.670763

**Published:** 2021-06-11

**Authors:** Raquel Freitas Zambonatto, Renata Nakata Teixeira, Sarah de Oliveira Poma, Eliane Borges da Silva, Mariana Mendes de Almeida, Gerson dos Santos Leite, Cesar Miguel Momesso dos Santos, Heloisa Helena de Oliveira Alves, Renata Gorjão, Tania Cristina Pithon-Curi, Celso R. F. Carvalho, Rui Curi, Adriana Cristina Levada-Pires

**Affiliations:** ^1^ Institute of Physical Activity Sciences and Sports, Interdisciplinary Post-graduate Program in Health Sciences, Cruzeiro do Sul University, Sao Paulo, Brazil; ^2^ Department of Physical Therapy, School of Medicine, University of Sao Paulo, Sao Paulo, Brazil

**Keywords:** athletes, leukocytes, cytokines, lipopolysaccharide, allergy, IgE

## Abstract

We collected peripheral blood from thirty-nine elite male endurance runners at rest (24 hours after the last exercise session) and used the Allergy Questionnaire for Athletes score and plasma specific IgE level to separate them into atopic and non-atopic athletes. Neutrophils obtained from atopic and non-atopic athletes were subsequently stimulated *in vitro* with fMLP (N-formyl-methionyl-leucyl-phenylalanine), LPS (lipopolysaccharide), or PMA (phorbol 12-myristate 13-acetate). Neutrophils from non-atopic runners responded appropriately to LPS, as evidenced by the production of pro (IL-8, TNF-α, and IL-6) and anti-inflammatory (IL-10) cytokines. Neutrophils from atopic elite runners exhibited lower responses to LPS stimulus as indicated by no increase in IL-1β, TNF-α, and IL-6 production. Neutrophils from non-atopic and atopic runners responded similarly to fMLP stimulation, indicating that migration function remained unaltered. Both groups were unresponsive to PMA induced reactive oxygen species (ROS) production. Training hours and training volume were not associated with neutrophil IgE receptor gene expression or any evaluated neutrophil function. Since non-atopic runners normally responded to LPS stimulation, the reduced neutrophil response to the stimuli was most likely due to the atopic state and not exercise training. The findings reported are of clinical relevance because atopic runners exhibit a constant decline in competition performance and are more susceptible to invading microorganisms.

**Graphical Abstract:**
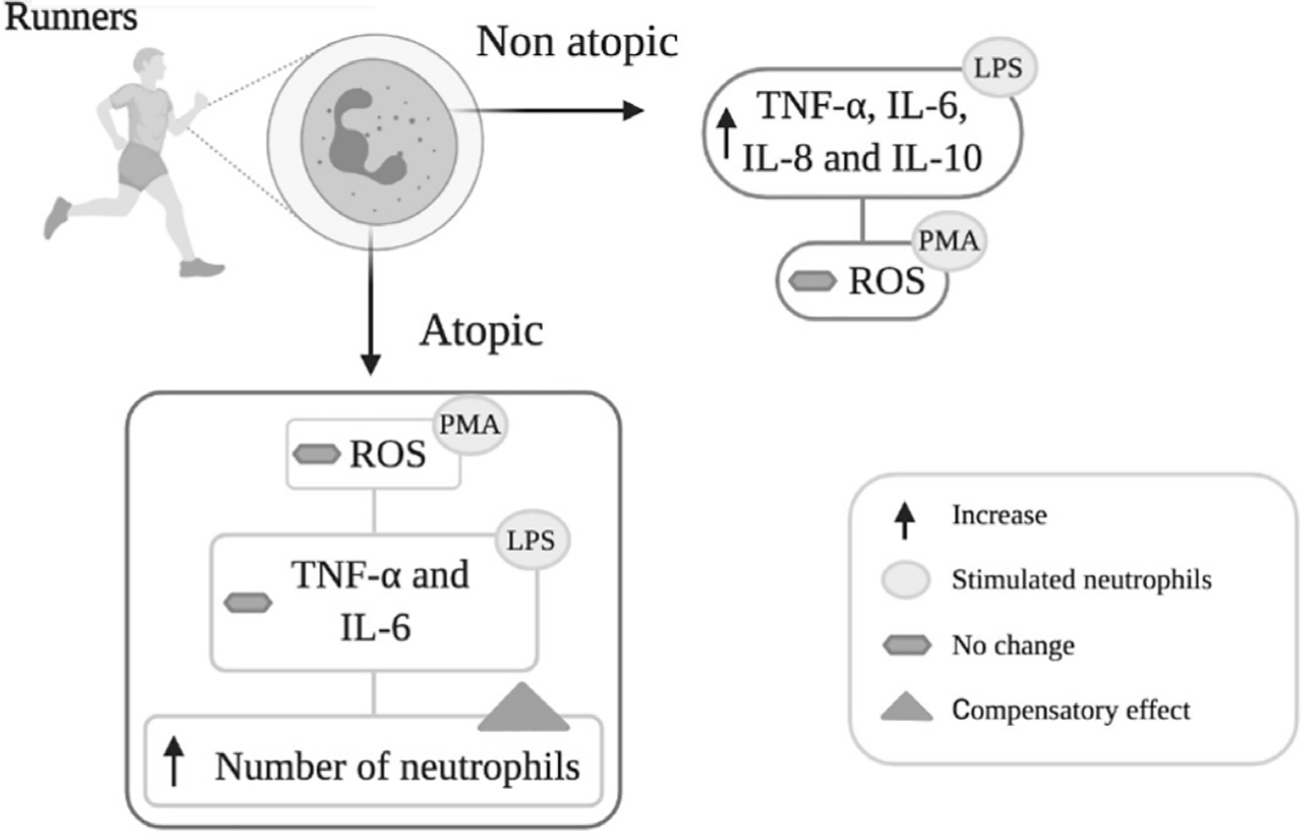
Neutrophils isolated from non-atopic runners responded to LPS stimulation by increasing cytokine (TNF-α, IL-6, IL-8 and IL-10) production, but PMA stimulation did not change ROS production. Neutrophils from the atopic group only exhibited increased IL-8 and IL-10 production in response to LPS stimulation and were also unresponsive to PMA. The reduction in neutrophil responsiveness to LPS in atopic runners was accompanied by an increase in total circulating neutrophil counts. The increase in the number of circulating neutrophils may reflect a compensatory response to attenuated neutrophil function.

## Introduction

Atopy is an immune response that induces immunoglobulin E (IgE) overproduction after exposure to an allergen (1). Although reported as a genetic condition, elite endurance sports athletes exhibit an increased prevalence of atopy (2–5). This heightened susceptibility is mainly due to the nasopharyngeal mucosa being repeatedly exposed to environmental irritants such as internal and external air pollution, pollen allergens, cold air, and variable humidity during exercise training (6–8). Indeed, Spence et al. reported upper respiratory tract infection symptoms in athletes, and only 5% of the episodes occurred due to bacterial infections (9). In addition, Bonini et al. demonstrated that the high prevalence of allergy and asthma was associated with recurrent upper respiratory tract infections (10).

The risk of asthma is higher in atopic long-distance runners (42-fold), speed and power athletes (25-fold), and swimmers (97-fold) (8). These results indicated that the respiratory symptoms are associated with an inflammatory condition rather than infectious agents. Previous studies reported that atopy is associated with the altered function of immune cells such as eosinophils, lymphocytes, mast cells, and neutrophils (11–13).

Neutrophils express three IgE receptors: FceRI, FceRII/CD23, and galectin-3 (12, 14), which modulate IgE-dependent mechanisms (14). Neutrophils play an essential role in the inflammatory process (15, 16). Following activation, these inflammatory cells migrate towards the inflammation site, where they engulf particulate materials *via* phagocytosis and produce reactive oxygen species (ROS) (17) to eliminate the pathogens and/or damaged tissue (18). Activated neutrophils also synthesize and release pro- and anti-inflammatory cytokines, coordinating the inflammatory process (19, 20) by recruiting other neutrophils, macrophages and/or lymphocytes. Higher levels of pro inflammatory cytokine production prolong the duration of inflammation (21).

Neutrophils can undergo apoptosis to control inflammation. Apoptosis indicators include DNA fragmentation, chromatin condensation, cell volume reduction, phosphatidylserine externalization, mitochondrial membrane depolarization, and apoptotic body formation. Before the membrane integrity and intracellular contents are lost, neutrophils undergoing apoptosis are removed by macrophages and other neutrophils, consequently reducing the inflammatory response (22, 23). Two classical apoptosis pathways exist in neutrophils, extrinsic and intrinsic (24, 25). Extrinsic apoptosis is triggered by the activation of membrane surface receptors, such as Fas, APO-2L, TRAIL, and TNF-α, which subsequently induce the activation of caspases 8 and 3 and lead to cell death. The intrinsic pathway involves losing mitochondrial membrane potential (26). In this situation, pro-apoptotic molecules such as Bax and Bak form heterodimers that migrate to the outer mitochondrial membrane and form pores, leading to the release of cytochrome c associated with Apaf-1 and pro-caspase 9, the formation of the apoptosome, and activation of caspase 9. This latter enzyme cleaves and activates caspase 3, resulting in cell death. Anti-apoptotic proteins such as Bcl-2 and Bcl-xL prevent Bax-induced pore formation and inhibit apoptosis. Thus, the Bax/Bcl-xL ratio controls cell survival (25, 27).

Neutrophils from patients, when stimulated with specific allergens, reduce the L-selectin expression (28), increase IL-8 production (29), and release myeloperoxidase, elastase, and defensin (12, 14), leading to clinical allergy symptoms.

Previously, our group detected delayed neutrophil activation in elite athletes after completing a competition. We also observed for the first time that neutrophils of half-ironman triathlon athletes undergo apoptosis immediately after a competition and found that this process is associated with an increase in plasma fatty acid levels (30). It is plausible that the higher frequency of atopy reported in elite endurance athletes may occur due to prolonged high-intensity exercise training, which may impair immune function (7). Although there are numerous reports of allergy and asthma in athletes, the effect of atopy on immune cells, such as neutrophils, has yet to be elucidated.

In the present study, we measured features of neutrophils from atopic and non-atopic elite endurance runners. For this purpose, we used the Allergy Questionnaire for Athletes score and plasma specific IgE levels to separate them into atopic and non-atopic athletes. The following clinical parameters were measured: blood leukocytes, red blood cells, hematocrit, hemoglobin, non-esterified free fatty acids (NEFAs), testosterone, and cortisol. Blood neutrophils were isolated from the athletes at rest 24 hours after the last exercise session. Neutrophils were treated with fMLP (N-formylmethionyl-leucyl-phenylalanine), a chemotactic factor (31–33) to measure cell migration, PMA (phorbol 12-myristate 13-acetate), an activator of PKC (34) to measure ROS production, or LPS (lipopolysaccharide), a toll-like receptor 4 agonist that activates NF-ƙB transcription factor (35, 36) to evaluate secretion of cytokines (IL-1β, TNF-α, IL-6, IL-8, and IL-10). The same parameters were measured in unstimulated neutrophils. Expressions of L-selectin (CD-62L) in cell surface, IgE receptors gene expression, and phagocytic capacity were measured in unstimulated neutrophils. Indicators of apoptosis (such as membrane integrity, DNA fragmentation, expressions of Fas (CD95), TNFR2 (CD120b), and TRAIL (CD253) in cell membrane surface, and Bax and Bcl-xL mRNA) were also determined in unstimulated neutrophils. The results from atopic and non-atopic runners were compared to find out atopy associated changes in neutrophil functioning.

## Materials and Methods

### Subjects

Thirty-nine male runners were recruited during the packet pick-up period that occurred the week before the most internationally prestigious Brazilian long-distance street race, “Corrida de São Silvestre”, held in the city of São Paulo, Brazil. The runners were informed about the aim of the study and signed the free and informed consent term. The inclusion criteria were previously defined (37) and included males competing at an elite level, based on the runners’ performance in the previous year. As previously described (7, 37), this study included runners who could run a 10 km race in <33 min; half-marathon in <1h and 10min and marathon in <2h and 30 min. Participants did not take supplements or medication since 15 days before the assessments. All evaluations were performed at rest, 24 hours after the last training session, and no longer than one month after the race. All study participants provided written informed consent before enrollment. The Research Ethics Committee of the School of Medicine, University of São Paulo approved the study (protocol number 20714).

### Experimental Design

Runners reported their training information, which was based on the week before the race. This information was collected to ensure that all athletes maintained similar training conditions, as previously described (4). All runners were evaluated in the morning from 07:00 AM to 09:00 AM to reduce the influence of the circadian cycles. Atopy was determined based on the Allergy Questionnaire for Athletes (AQUA) score and plasma specific IgE levels (38). Athletes with AQUA scores ≥ 5 and plasma specific IgE levels ≥ 0.35 kU/L were classified as atopic. Non-atopic included athletes with AQUA scores < 5 or plasma specific IgE levels < 0.35 kU/L, or both, as previously described (37).

### Characterization of Atopy

The AQUA questionnaire was specifically developed for assessing allergic symptoms in athletes, and it was validated in Portuguese. The copyright holders granted permission to use the AQUA survey. Briefly, this questionnaire comprises 25 items related to allergic symptoms, family history of allergies, suspicion of allergies, and the use of allergy medicines. Questions 4 to 16 (except for question number 14) received a score. Responses were related to objective documentation of allergies (e.g., positive skin tests and/or specific IgE antibodies to at least one allergen). The positive likelihood ratio (LR+) was calculated based on the following formula: LR+ = sensitivity/(1 − specificity). The LR+ indicates the odds of the disease increasing when a test is positive. Each question then receives a score based on the LR+ value and, according to a scale, derived from the guidelines for interpreting LR. In the present study, the sum of these question scores was used to classify athletes with allergies. Those with scores of ≥5 had a high risk of developing a disease, as previously described (38, 39).

Plasma specific IgE levels were determined using the Phadiatop method (Phadia 100, Thermo-Scientific, Phadia AB, Uppsala, Sweden) as previously described (38). A panel, including the most commonly inhaled allergens, such as house dust mites (Dermatophagoides pteronyssinus, Dermatophagoides farina, and Blomia tropicalis), cats, and cockroaches were analyzed. Sensitization was considered when the plasma specific IgE levels was ≥0.35 kU/L (2, 6). We used the same protocol in a previous study (7).

### Training Parameters

Runners reported training experience, number of training sessions per week, time spent training (in hours), and training volume (in km per week). These questions have been previously used (4, 14, 37) and were based on the week before data collection to ensure that all athletes had similar training conditions (4).

### Plasma Separation

Blood samples (20 mL) were collected from the antecubital vein of the runners at rest into vacuum tubes containing 0.004% ethylenediaminetetraacetic acid (EDTA). The plasma was separated from the whole blood by centrifuging the samples at 400 × g for 10 min. The upper phase, containing the plasma, was removed, and stored at –80°C. The samples were submitted to one centrifugation before being stored. For the assays, the samples were thawed and submitted to another centrifugation.

### Clinical Parameters Measurements

CDA-Diagnostic Laboratory (São Paulo, Brazil) performed blood leukocytes, red blood cell counting, hematocrit, hemoglobin using the Sysmex XT 2000 equipment (Kobe, Japan). Plasma creatine phosphokinase (CPK) activity was determined by using Cobas C-311 analyzer (Roche, CA, USA). Plasma concentration of non-esterified free fatty acids **(**NEFAs) was determined with the NEFA HR Kit (Wako Chemical, Neuss, Germany). This enzymatic and colorimetric assay was performed following the manufacturer’s instructions, as previously reported (30, 40). Testosterone and cortisol were evaluated using a high-affinity monoclonal antibody that binds specifically against testosterone (41) or cortisol (42), following the manufacturer’s instructions (Elecsys, Roche Diagnostics, Penzberg, Germany).

### Neutrophil Isolation

Blood samples were collected in vacuum tubes containing an anticoagulant (0.004% EDTA) from the antecubital vein of the runners at rest. Neutrophils were obtained from whole blood diluted in phosphate-buffered saline (PBS, 1:1; pH 7.4 containing 100 mmol/L CaCl_2_, 50 mmol/L MgCl_2_) and carefully layered on histopaque (d = 1.077) gradient (43, 44). The tubes were then centrifuged at 400 x g and 4°C for 30 min. The supernatant, rich in mononuclear cells, was separated. Neutrophils were prepared from the inferior sediment, and 10 mL lyse solution, containing 150 mmol/L NH_4_Cl, 10 mmol/L NaHCO_3_, 0.1 mmol/L ethylenediaminetetraacetic acid (EDTA), pH 7.4, were added to promote lysis of contaminating erythrocytes. The preparation was homogenized and maintained in ice for 10 min to allow erythrocyte lysis. Afterward, the tubes were centrifuged at 400 x g and 4°C for 10 min, and this procedure was repeated to reduce residual erythrocyte. Cells were washed once with PBS. Neutrophils were then counted in a Neubauer chamber in an optical microscope (Carl Zeiss, Jena, Germany), according to the protocol used in our previous study (30, 45).

### Measurements in Neutrophils in the Absence or Presence of FMLP, PMA, or LPS

#### Neutrophil Migration

Neutrophil migration was evaluated in 24-well disposable chemotactic plates (Neuro Probe) according to the manufacturer’s instructions, in the absence and presence of 10 nmol/L fMLP (Sigma Aldrich). Briefly, flat-bottomed chambers were filled with the chemotactic agent - fMLP in phosphate-buffered saline (PBS) containing 0.01% albumin. Chemotactic membranes with a pore size of 5 μm were fixed to the filter seat. The chamber assembly with neutrophils (1 × 10^6^ per mL) was incubated in a humidified 5% CO_2_ atmosphere at 37°C for 60 minutes. After incubation, the chamber was disassembled, and the cells that migrated, crossing the plasma membrane, were counted directly in the Neubauer chamber in an optical microscope (Carl Zeiss). Chemotactic responses were defined as the mean number of migrated neutrophils. The same assay was used in our previous study (46).

#### Reactive Oxygen Species (ROS) Production

Neutrophils (2 × 10^6^/mL) were incubated in the absence and presence of 80 ng/mL PMA (Sigma Aldrich) for 30 min, and ROS production was measured using dihydroethidium (1 micromole/L) in a BD-Accuri flow cytometer (Becton Dickinson), as described in our previous study (47).

#### Measurement of Cytokines

Isolated neutrophils (1 × 10^6^/mL) were suspended in RPMI 1640 medium supplemented with 10% fetal bovine serum, cultured at 37°C in a 5% CO_2_ atmosphere, and incubated in the absence and presence of 5 μg/mL LPS (Sigma Aldrich). After 18 hours of cell culture, the supernatant was collected and stored at −80°C until the cytokine measurements were performed. The supernatant levels of IL-1β, TNF-α, IL-6, IL-8, and IL-10 were determined using the BD Human Inflammatory Cytokine cytometric Bead Array Kit or the BD Accuri cytometer according to manufacturer’s instructions (Becton Dickinson). Results were analyzed using the BD CSampler software (Becton Dickinson). Data acquisition was performed with the BD-AccuriC6 software, and the cytokine concentrations were determined using the FCAP software v.3.0. According Becton Dickinson the limits of detection for these cytokines are: 7.2 pg/mL for IL-1β, 3.7 pg/mL for TNF-α, 2.5 pg/mL for IL-6, 3.6 pg/mL for IL-8, and 3.3 pg/mL for IL-10. The statistical analysis of the samples that showed values lower than the minimum detected concentration (indicating low production) was performed considering the intermediate point between zero and the lowest value.

### Measurements in Unstimulated Neutrophils

#### Expression of the Surface Molecule L-Selectin (CD62L)

**N**eutrophils (1 × 10^6^/mL) were incubated with an anti-CD62L antibody conjugated to fluorescein isothiocyanate (CD62L-FITC, Becton Dickinson) for 30 minutes in the dark at room temperature. The expression of CD62L on the surface of the neutrophils was analyzed by flow cytometry, as previously described (47).

#### Phagocytosis Capacity

Neutrophil phagocytosis assay was carried out using zymosan particles (Zymosan A, which is an insoluble large polysaccharide fraction from yeast cell walls). The zymosan particles were suspended in PBS to achieve a concentration of 1 × 10^7^ particles/mL. For opsonization, it was used the proportion 1:1 zymosan particles mixed with normal human serum and incubated for 40 min at 37°C. Opsonized zymosan particles were centrifuged at 400 x g for 10 min in PBS and suspended in RPMI 1640 medium for the phagocytosis assay. Neutrophils (2 x 10^6^) were incubated in 1mL of RPMI 1640 medium containing opsonized zymosan for 40 min at 37°C. Neutrophils were diluted (1:10) in a 1% crystal violet solution (prepared with acetic acid) and counted in a Neubauer chamber using an optical microscope (Carl Zeiss). Phagocytosis was expressed as the percentage of cells that had phagocytosed more than three particles (as determined in an optical microscope at 400 x magnification), as previously described (48).

#### Plasma Membrane Integrity Assay

Neutrophils (1 × 10^6^/mL) were re-suspended in 100 µL PBS and 50 µL of 20 µg/mL propidium iodide (PI) solution (in PBS) was then added. The membrane integrity was determined by using a BD-Accuri flow cytometer (Becton Dickinson). Neutrophils (1 x 10^6^ cells) were resuspended in 100 µL of PBS, and 50 µL of propidium iodide (PI) solution (20 µg/mL in PBS) were then added. Propidium iodide is a highly water-soluble fluorescent compound that cannot pass through intact membranes and is generally excluded from viable cells. It binds to DNA by intercalating between the bases with little or no sequence preference. Fluorescence was measured using the FL2 channel (orange-red fluorescence = 585/42 nm). Ten thousand events were analyzed per experiment. Results are expressed as the percentage of cells that present intact membranes. The same procedure was described in our previous study (47).

#### DNA Fragmentation Assay

DNA fragmentation was measured using a BD-Accuri flow cytometer (Becton Dickinson), fluorescence was measured using the FL2 channel (orange-red fluorescence = 585/42 nm). Ten thousand events were analyzed per experiment. Analyses were performed using BD CSampler™ software (Becton Dickinson). Results are expressed as the percentage of cells that present fragmented DNA, as previously described (49) and in our previous study (4).

#### Expression of Apoptotic Membrane Surface Molecules

The surface expression of TRAIL (CD253), TNFR2 receptor (CD120b), and Fas (CD95) (Becton Dickinson) was analyzed using a BD Accuri flow cytometer (Becton Dickinson). Neutrophils (1 × 10^6^/mL) were incubated with antibodies conjugated to allophycocyanin (CD253-APC), phycoerythrin (CD120-PE) or FAS (CD95-FAS) for 30 minutes in the dark at room temperature. The fluorescence was determined at the following wavelengths: 660/20 nm (APC), 695/40 nm (PE), or 530/30 nm (FITC), and ten thousand events per sample were acquired in the histograms. Data analysis was performed using the BD CSampler software (Becton Dickinson). Results are expressed as the mean fluorescence. This assay was performed as previously described (47).

#### Gene Expressions of Bax, Bcl-xL, and IgE Receptor Measured by Quantitative Real-Time Polymerase Chain Reaction (qPCR)

Total RNA was obtained from neutrophils (1 × 10^7^) using the guanidine isothiocyanate extraction method with TRIzol Reagent (Invitrogen, Carlsbad, CA, USA) and isolated using the RNeasy mini kit (Qiagen, Hilden, Germany). The purity was assessed by the 260/280 nm absorbance ratio, and the quantity was calculated based on the absorbance at 260 nm. The cDNA was synthesized from 1.0 μg of total RNA using the High Capacity cDNA Reverse Transcription Kit (Invitrogen). Real-time PCR analysis was performed using the SyBR Green JumpStart kit (Sigma Aldrich, MO, USA) and QuantStudio^®^ 3 Real-Time PCR system (Thermo Fisher Scientific, Massachusetts, EUA). Duplicate analysis was performed on all samples. Each gene melting curve (including housekeeping and target genes) was first analyzed by QuantStudio Design & Analysis Software (Thermo Fisher Scientific, Massachusetts, EUA) to ensure the assay specificity. Moreover, negative controls were used to ensure the absence of contamination. The standardization of the reaction was carried out utilizing efficiency curves with efficiency percentages between 90 and 110%, slopes close to -3.33 and R2 values greater than 0.99. Levels of the all mRNAs detected in each samples were reported as the relative expression with regard to the 2 controls [YWHAZ (Tyrosine 3-Monooxygenase/Tryptophan 5-Monooxygenase Activation Protein Zeta) and SDHA (Succinate Dehydrogenase Complex Flavoprotein Subunit A)], as indicated after analysis in geNorm software); and using the geometric mean of Ct for ΔCt analysis (ΔCt = Ct of target gene – Ct geometric mean of controls) (50). The relative expression of mRNAs was calculated by the 2^−ΔΔCT^ method prior to the statistical analysis (51). The same procedure was employed in a previous study (52). The annealing temperature used for all genes investigated was 60°C. The sense and antisense primers, and fragment lengths are presented in **Table 1**.

**Table 1:** Primer sequences for quantifying Ywhaz, SDHA, Bax, Bcl-xL and IgE receptor gene expression analyses.

	Primer Forward	Primer Reverse
Ywhaz	TGCAAAGACAGCTTTTGATGAAGCC	GCAGACAAAAGTTGGAAGGCCGG
SDHA	TGATGCTGTGTGCGCTGCAG	TCCAGAGTGACCTTCCCAGTGCCA
Bax	GCATCCACCAAGAAGCTGAGC	CACAAAGATGGTCACTGTCTGCC
Bcl-xL	AAACCCCAAGTCCTCCTTGC	CCAATACGCCGCAACTCTTG
IgE receptor	TACCAGATACAGCCCGTCCT	AATACATGGCGGTGTCCGAG

### Sample Size Estimation

A ROS production greater than 100% and DNA fragmentation greater than 10% was used to test the hypothesis of a difference in ROS production and DNA fragmentation between groups. Levada-Pires et al. (30) reported a standard deviation of around 11.1 (relative units) for ROS production and 3% for DNA fragmentation in non-stimulated neutrophils from athletes at rest (30). A sample size of at least 14 runners was required using the alpha and power set at 0.05 and 0.8, respectively.

### Statistical Analyses

Quantitative variables are presented as the mean ± standard deviation (text and **Table 2**) or median and quartile interval as the log-natural (figures). Depending on the normality assumption as determined with the Kolmogorov-Smirnov test, we used the Student’s t-test or the Mann-Whitney test for comparisons of quantitative variables between two groups. Spearman correlation was used to investigate relationships between the quantitative variables. Adjustment of the generalized linear regression model was performed with variables dependent on interleukins and explanatory variables (independent), the number of training sessions, and atopy. The level of significance was 0.05. The data analysis was performed using the SPSS software (Statistical Package for the Social Sciences) for Windows, version 19 and R core 3.4.

**Table 2:** Age, anthropometric characteristics, clinical parameters and training information from non-atopic and atopic runners at rest.

	Non-atopic (n = 28)	Atopic (n = 11)	p-value
**Age**, years	32.9 ± 5.6	27.9 ± 6.4	0.06
**Anthropometric characteristics**			
Weight, kg	63.7 ± 5.3	62.71 ± 4.0	0.56
Height, m	1.73 ± 0.06	1.77 ± 0.05	0.12
**Clinical parameters**			
Leukocytes, x 10^3^/μL	4909 ± 1069	6025 ± 1445	0.01*
Neutrophil, x 10^3^/μL	2475 ± 872.9	3579 ± 1354	0.004**
Lymphocyte, x 10^3^/μL	1849 ± 531.4	1871 ± 618.5	0.96
Monocytes, x 10^3^/μL	442.2 ± 150.6	426.4 ± 95.1	0.96
Basophil, x 10^3^/μL	13.4 ± 8.1	18.9 ± 12.4	0.08
Eosinophil, x 10^3^/μL	129.6 ± 140.1	128.4 ± 92.1	0.63
Red blood cells, x 10^3^/μL	5.09 ± 0.4	4.96 ± 0.41	0.25
Hemoglobin, g/dL	14.9 ± 0.9	15.1 ± 1.41	0.7
Hematocrit, %	43.9 ± 2.6	44.8 ± 3.3	0.38
NEFAs, mM	0.14 ± 0.08	0.12 ± 0.04	0.79
Creatine phosphokinase, U/L	329.6 ± 195	361.5 ± 205.9	0.58
Cortisol, μg/dL	0.41 ± 0.21	0.39 ± 0.13	0.77
Testosterone, ng/dL	428.5 ± 105.3	443 ± 74.82	0.84
**Training information**			
Training experience, years	10.60 ± 6.26	7.81 ± 4.14	0.22
Number of training sessions per week	7.35 ± 2.24	9.27 ± 2.61	0.02*
Hours of training per week	12.2 ± 3.9	14.6 ± 5.2	0.18
Volume of training, km per week	97.6 ± 23.0	116.5 ± 46.1	0.09

The data were parametrically distributed and are expressed as the mean ± SD. kg, kilogram; m, meter. In bold: **p < 0.01, *p < 0.05 between atopic and non-atopic athletes.

## Results

The frequency of atopy in the elite athletes was determined to be 28.2% (n=11), whereas 71.8% of the athletes (n=28) were classified as non-atopic. Plasma specific IgE levels and AQUA^©^ questionnaire scores were higher in the atopic athletes when comparing to non-atopic 10.7 ± 13.0 *vs.* 2.7 ± 7.0 Ku/L and 10.8 ± 5.4 *vs.* 3.5 ± 4.11 score, respectively (p˂0.001). The frequency of atopy in the studied group was similar to its overall epidemiology in other countries such as Australia and Turkey-Antalya (53, 54).

No difference was detected in the age and anthropometric characteristics of atopic and non-atopic athletes (**Table 2**). The atopic group presented a greater number of training sessions per week than the non-atopic group (p=0.02; **Table 2**). An association was identified between the AQUA score and the number of training sessions per week (r=0.35; p=0.03). There was no difference between groups in the clinical parameters measured: blood numbers of lymphocytes, eosinophils, monocytes, basophils, and red blood cells, hematocrit, hemoglobin, plasma levels of free fatty acids, cortisol, testosterone, and creatine phosphokinase activity. The atopic group exhibited 1.4-times and 1.2-times higher number of blood total leukocytes and neutrophils than the non-atopic group, respectively (**Table 2**).

**Figure 1:**
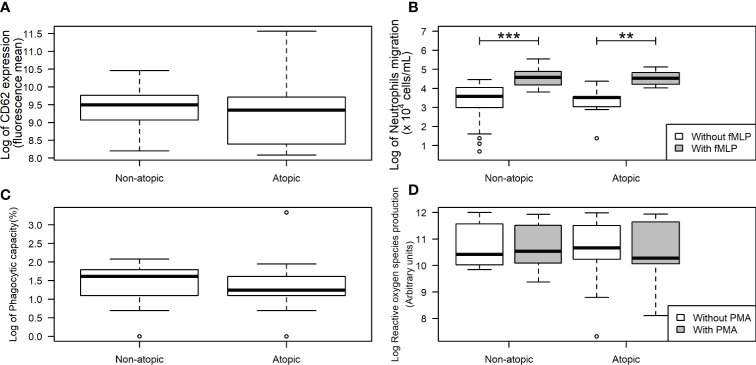
L-Selectin expression and functions in neutrophils from non-atopic and atopic runners. Neutrophils were obtained from the bloodstream of non-atopic and atopic runners at rest, and L-selectin (CD62L) expression on the neutrophil surface **(A)**, migration **(B)**, phagocytic capacity **(C)**, and reactive oxygen species production **(D)** were assessed. The results are presented as medians (middle lines). The 25–75% confidence intervals (closed boxes) and the 5–95% confidence intervals (lower and upper lines, respectively) are indicated. ***p < 0.0001 when compared to neutrophils (in the absence of fMLP) of non-atopic runners. **p < 0.01 when compared to neutrophils (in the absence of fMLP) of atopic runners.

**Figure 2:**
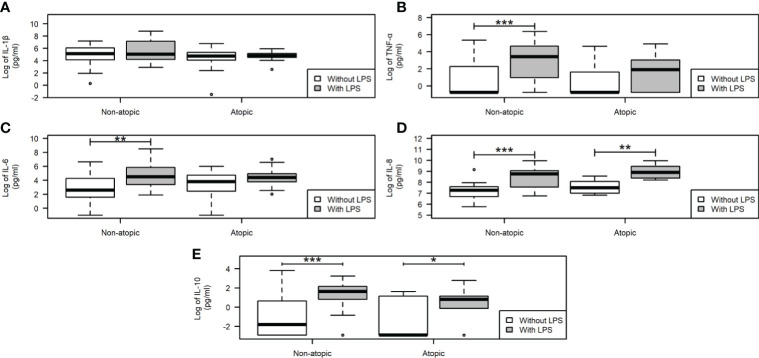
Cytokine production by neutrophils from non-atopic and atopic runners. Production of proinflammatory IL-1β **(A)**, TNF-α **(B)**, IL-6 **(C)**, IL-8 **(D)**, and the anti-inflammatory IL-10 **(E)** cytokines. After incubation, the supernatant was removed, the cytokine secretion was measured by CBA upon stimulation with 5 μg/mL LPS for 18 h, and data were acquired by flow cytometry using the BD Accuri software and analyzed using the FCAP software (Becton Dickinson, San Juan, CA, USA). The results are presented as medians (middle lines). The 25–75% confidence intervals (closed boxes) and the 5–95% confidence intervals (lower and upper lines, respectively) are indicated. *p < 0.05 when comparing neutrophils in the absence of LPS stimulus. *p < 0.05 when comparing neutrophils in the absence of LPS stimulus. **p < 0.01 and ***p < 0.0001.

Neutrophils from non-atopic and atopic runners responded similarly to fMLP stimulation, with both groups exhibiting an increase in migration (**Figure 1B**). ROS production by isolated neutrophils was equivalent in the absence and presence of PMA stimulation for both groups (**Figure 1D**). The production of IL-1β by neutrophils from both groups remained unchanged, even after the LPS stimulation (**Figure 2A**). However, LPS stimulation augmented the production of pro- (TNF-α, IL-6, and IL-8) and anti-inflammatory cytokines (IL-10) in neutrophils from non-atopic athletes. Neutrophils from atopic athletes only exhibited increased production of IL-8 and IL 10. The levels of the TNF-α and IL-6 were not different between the groups (**Figures 2B–E**). Unstimulated neutrophils from both groups were compared and there was no significant difference in L-selectin (CD62L) expression on the cell surface (**Figure 1A**), migratory (**Figure 1B**) and phagocytic capacity (**Figure 1C**), and production of pro and anti-inflammatory cytokines (**Figures 2A–E**).

**Figure 3:**
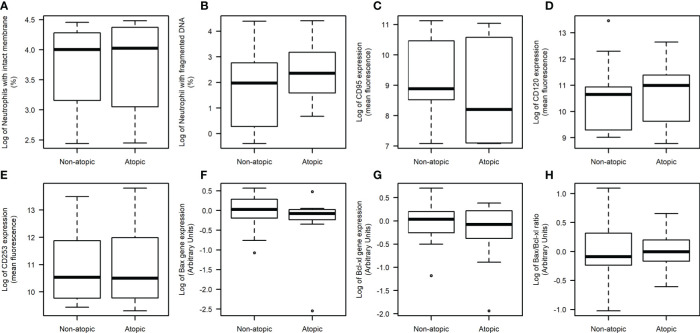
Neutrophil death evaluation in non-atopic and atopic runners. Freshly obtained cells were isolated from the blood of non-atopic and atopic runners at rest. Percentage of neutrophils with an intact membrane **(A)** and DNA fragmentation **(B)**. The expression of Fas (CD95) **(C)**, TNFR2 (CD120b) **(D)** and TRAIL (CD253) **(E)** receptors on the neutrophil surface, as determined by flow cytometry. Ten thousand events were evaluated per experiment. The gene expression levels of Bax **(F)**, Bcl-xL **(G)** and the Bax/Bcl-xL ratio **(H)** as determined by qPCR. The expression levels were normalized using two housekeeping genes (YWHAZ and SDHA), as indicated by the geNorm program (Vandesompele et al., 2002). The data were analyzed by the 2^-ΔΔCT^ method. The results are presented as medians (middle lines). The 25–75% confidence intervals (closed boxes) and the 5–95% confidence intervals (lower and upper lines, respectively) are indicated.

Apoptotic parameters were evaluated in unstimulated neutrophils. The proportion of neutrophils with an intact membrane and DNA fragmentation was similar between atopic and non-atopic runners (**Figures 3A, B**), indicating that the apoptosis was not compromised by atopy. A positive linear correlation between DNA fragmentation and plasma IgE concentration (r=0.33; p=0.04) was detected. The expression levels of Fas (CD95), TRAIL (CD253), and TNFR2 (CD120b) receptors in the neutrophil plasma membrane were also similar between groups (**Figures 3C–E**, respectively). Expressions of Bax and Bcl-xL genes in neutrophils were not different between atopic and non-atopic runners (**Figures 3F, G**) and so the Bax/Bcl-xL ratio (**Figure 3H**). There was also no difference in the IgE receptor gene expression in unstimulated neutrophils from both groups (date not shown).

The correlation analyses between the number of weekly training sessions and neutrophil function measurements revealed a weak positive correlation between the number of weekly sessions and the production of IL-8 (r=0.36; p=0.016) and IL-10 (r=0.31; p=0.04) in non-stimulated neutrophils. A generalized linear regression model using the number of weekly sessions and atopy as independent explanatory variables and the interleukin levels (IL-8 and IL-10) as dependent variables was determined. After adjusting the multivariate model to remove the influence of aberrant points in the data, none of the variables presented statistical significance. The number of training sessions and atopy did not significantly correlate with IL-8 production by unstimulated and LPS-stimulated neutrophils. On the other hand, these two variables (atopy and number of training sessions) significantly correlated with IL-10 production. The unstimulated and LPS-stimulated neutrophils from the atopic group produced 0.96 (p=0.007) and 4.98 (p=0.003) pg/mL less IL-10 than the non-atopic group, respectively. Each additional training session per week increased the production of IL-10 by 0.41 (p=0.036) and 0.81 (p=0.01) pg/mL in unstimulated and LPS stimulated neutrophils, respectively.

## Discussion

In the present study, we report for the first time the association between atopy and neutrophil functions in elite runners. There was no difference in the clinical parameters between the two groups investigated, demonstrating that the physiological condition was similar. Notably, we found that neutrophils isolated from atopic runners did not adequately respond to PMA and LPS stimulation, as evidenced by the lack of stimuli induced an increase in ROS and cytokine (IL-1β, TNF-α, and IL-6) production. These alterations might impair an efficient control of inflammation and antigen response in atopic athletes.

L-Selectin (CD62L) upregulation is essential for neutrophil adherence to the endothelium and migration to the inflammation site (55). Neutrophils from patients with allergic rhinitis have a high CD62L expression (56), whereas others reported down-regulation in allergic patients challenged with specific allergens (28, 56). The expression of CD62L was increased 72 hours after a marathon competition (45). CD62L expression was counterbalanced by the atopy and exercise and may explain why we did not observe differences between both groups. We previously reported reduced neutrophil phagocytosis in athletes compared to non-athletes (30). Herein, neutrophil phagocytosis capacity was not different between non-atopic and atopic athletes. These results suggest that atopy does modify neutrophil phagocytosis capacity.

ROS production plays a crucial role in neutrophil cytolytic activity (30). However, ROS overproduction by neutrophils leads to cell death and tissue damage (57). PMA stimulation did not increase ROS production in neutrophils from atopic and non-atopic runners at rest. We reported similar observations in neutrophils from athletes after a competition (30). Exercise training per se raises neutrophil ROS production mimicking a drug stimulating effect that does not increase any further, even *in vitro* stimulation.

LPS-stimulated neutrophils from non-atopic runners exhibited increased pro inflammatory (TNF-α, IL-8, and IL-6) and anti-inflammatory (IL-10) cytokine release. On the other hand, LPS-stimulated neutrophils from atopic runners only exhibited augmented IL-8 and IL-10 production. The increased IL-8 production may play a role in maintaining the migration capacity and CD62L expression in atopic and non-atopic runners. IL-8 activates neutrophils and up-regulates the expression of adhesion molecules (58). Neutrophil migration is known to increase IL-8 production to recruit more neutrophils (29, 59).

Allergies modulate the function of neutrophils through IgE-dependent mechanisms. Monteseirin et al. (29) reported that IgE-dependent IL-8 production involved ROS and calcium/calcineurin. Neutrophils from allergic patients stimulated with LPS exhibited NF-κB activation, increasing IL-8 and thromboxane A2 production (29). Monocytes from atopic patients have an increase in the IL-10 production after LPS-stimulation when compared with healthy subjects (60). Despite this data, there is little information about the production of other pro and anti-inflammatory cytokines in studies investigating allergy effects on neutrophil function (12, 29, 61).

Interestingly, the reduction in neutrophil responsiveness to LPS in atopic runners was accompanied by an increase in total circulating neutrophil counts. The increase in circulating neutrophils may reflect a compensatory response to attenuated neutrophil function (62).

High-intensity exercise induces neutrophil apoptosis and impairs neutrophil function (30, 48, 63). Increased neutrophil apoptosis was reported in resting runners after intense exercise (45, 64). In our study, the apoptotic parameters were not altered when neutrophils of non-atopic and atopic groups were compared at rest. However, there was a positive correlation between plasma specific IgE levels and neutrophil DNA fragmentation.

The study would benefit by sampling also immediately after a competition. Further studies to address this issue and the inclusion of non-athletes for comparison will help elucidate the underlying mechanisms involved in atopy-induced hyporesponsive in neutrophils from atopic athletes herein reported.

We previously described that atopic endurance athletes exhibited a more significant lymphocyte mediated Th1 response than non-atopic athletes and proposed that the reduced Th1/Th2 ratio in athletes could partially impair the immune system homeostasis and may account for the increased occurrence of atopy in the athletes (37).

The modulation of NF-κB and p38 MAPK activation may account for the reduced cytokine production observed in LPS-stimulated neutrophils from atopic runners. The inhibition of p38 leads to a decrease in both IL-6 and TNF-α production (65). It is also possible that changes in cytokine release from the leukocytes after exposure to the stimulus are associated with a sequence polymorphism in the cytokine promoters, as shown with TNF-α and IL-1*β* release (66, 67). Such polymorphisms can affect translational initiation, mRNA stability, and polyadenylation (68). Since only a few studies have investigated neutrophil cytokine production in allergic athletes, it would be interesting to evaluate if atopy is associated with sequence polymorphisms in pro inflammatory cytokine promoters.

The changes we reported in neutrophil function might be associated with an impaired inflammatory response in atopic runners. Although further studies with a larger population, including non-athletes, are required to confirm the present results, the findings reported are of clinical relevance because atopic runners exhibit a constant decline in competition performance and are more susceptible to invading microorganisms.

## Data Availability Statement

The raw data supporting the conclusions of this article will be made available by the authors, without undue reservation.

## Ethics Statement

The studies involving human participants were reviewed and approved by Universidade de Sao Paulo (protocol number 20714). The patients/participants provided their written informed consent to participate in this study.

## Author Contributions

RZ participated in the recruitment of volunteers, performed blood collection from athletes and blood processing, isolation of plasma samples, determined neutrophils function and death, analyzed data, wrote manuscript, and prepared tables and figures. RT participated in the recruitment of volunteers, blood collection, analyzed plasma cortisol concentration and characterized athletes by determination of salivary IgE concentration, and analysis of AQUA questionnaire. ES, MA, and SP participated in blood processing, determined neutrophils function and death, and wrote part of the manuscript. GL participated in the recruitment of volunteers and characterization of athletes. CS and HA participated in the blood processing, isolation of plasma samples, storing, and further laboratory analysis. RG performed and analyzed the cytokines production by neutrophils and discussed results. TP-C discussed the results, wrote, and reviewed the manuscript. CC and RC participated in the planning and development of this study, discussed the results, and reviewed manuscripts. AL-P conceived, planned, and supervised the study, analyzed the data, discussed the results, wrote, and reviewed the manuscript. AL-P and CC were ahead of funding acquisition for this study. All authors contributed to the article and approved the submitted version.

## Funding

This work is supported by research funding from Fundação de Amparo à Pesquisa do Estado de São Paulo (FAPESP), Conselho Nacional de Desenvolvimento Científico e Tecnológico (CNPq), and Coordenação de Aperfeiçoamento de Pessoal de Nível Superior (CAPES) (99999.006711/2015-04).

## Conflict of Interest

The authors declare that the research was conducted in the absence of any commercial or financial relationships that could be construed as a potential conflict of interest.
